# Hunger and Appetite

**Published:** 1882-12

**Authors:** 


### HUNGER AND APPETITE.

Dr. Fournié, the French physiologist, distinguishes between hunger and appetite by describing the former as a general desire for food, no matter of what kind, while appetite is the feeling of pleasure which results from the gratification of that desire. This is proved by the fact that often, when we are not hungry, appetite comes while we are eating or at the mere sight and smell of some favorite dish. The question as to where the seat of the feeling of hunger is has been much discussed by physiologists. Leven asserts that it is not known at all, while Longet and Schiff believe that it is diffused through the whole body; but this latter view is disproved by the fact that in some diseases people waste away without ever having the slightest feeling of hunger. Dr. Fournié's theory is this: When meal-time arrives the glands of the stomach become filled and distended, and ready to accomplish their function of digesting the food. But if food is not introduced, they remain in this distended condition, and the result is the uneasy feeling we call hunger. Excellent proof of this theory is afforded by the habit of some Indians of eating clay to appease hunger. The introduction of the clay is followed by the discharge of the glands, and the sensation of hunger is arrested.

